# An In-Vitro Experimental Investigation of Oscillatory Flow in the Cerebral Aqueduct

**DOI:** 10.21203/rs.3.rs-2757861/v1

**Published:** 2023-04-03

**Authors:** Stephanie Sincomb, Francisco Moral-Pulido, Obed Campos, Carlos Martínez-Bazán, Victor Haughton, Antonio L. Sánchez

**Affiliations:** 1 Department of Mechanical and Aerospace Engineering, University of California- San Diego, La Jolla, US.; 2 Departamento de Ingeniería Mecánica y Minera, Universidad de Jaén, Jaén, Spain.; 3 Andalusian Institute for Earth System Research, Universidad de Jaén, Jaén, Spain.; 4 Departamento de Mecánica de Estructuras e Ingeniería Hidráulica, Universidad de Granada, Granada, Spain.; 5 Andalusian Institute for Earth System Research, Universidad de Granada, Granada, Spain.; 6 School of Medicine and Public Health, University of Wisconsin, Madison, US.

**Keywords:** Cerebrospinal fluid dynamics, Transmantle pressure, Normal pressure hydrocephalus

## Abstract

**Background:**

The cerebrospinal fluid filling the ventricles of the brain moves with a cyclic velocity driven by the transmantle pressure, or instantaneous pressure difference between the lateral ventricles and the cerebral subarachnoid space. This dynamic phenomenon is of particular interest for understanding ventriculomegaly in cases of normal pressure hydrocephalus (NPH). The magnitude of the transmantle pressure is small, on the order of a few Pascals, thereby hindering direct *in vivo* measurements. To complement previous computational efforts, we perform here, for the first time, *in vitro* experiments involving an MRI-informed experimental model of the cerebral aqueduct flow.

**Methods:**

Dimensional analysis is used in designing a scaled-up model of the aqueduct flow, with physical similarity maintained by adjusting the flow frequency and the properties of the working fluid. High-resolution MRI images are used to generate a 3D-printed anatomically correct aqueduct model. A programmable pump is used to generate a pulsatile flow rate signal measured from phase-contrast MRI. Extensive experiments are performed to investigate the relation between the cyclic fluctuations of the aqueduct flow rate and the transmantle pressure fluctuation over the range of flow conditions commonly encountered in healthy subjects. The time-dependent pressure measurements are validated through comparisons with predictions obtained with a previously derived computational model.

**Results:**

Parametric dependences of the pressure-fluctuation amplitude and its phase lag relative to the flow rate are delineated. The results indicate, for example, that the phase lag is nearly independent on the stroke volume. A simple expression relating the mean amplitude of the interventricular pressure difference (between third and fourth ventricle) with the stroke volume of the oscillatory flow is established.

**Conclusions:**

MRI-informed in-vitro experiments using an anatomically correct model of the cerebral aqueduct and a realistic flow rate have been used to characterize transmantle pressure. The quantitative results can be useful in enabling quick clinical assessments of transmantle pressure to be made from noninvasive phase contrast MRI measurements of aqueduct flow rates. The scaled-up experimental facility provides the ability to conduct future experiments specifically aimed at investigating altered CSF flow and associated transmantle pressure, as needed in connection with NPH studies.

## Introduction

The cerebrospinal fluid (CSF) fills the cerebral ventricles and the subarachnoid space (SAS) surrounding the brain and the spinal cord. Its motion has been studied and modeled extensively [1, 2], partly because of its role in key physiological functions associated with the transport of hormones, nutrients, and neuroendocrine substances [3–5] and partly because of its involvement in the development of different pathological conditions related to abnormal flow behavior, such as normal pressure hydrocephalus (NPH) [6, 7].

In addition to the slow steady flow existing between the ventricles, where CSF is produced by the choroid plexus, and the cerebral SAS, where CSF is primarily absorbed [8], there exists a much faster oscillatory motion driven by the spatial pressure differences induced by the cardiac and respiratory cycles [1,2,9–11]. In the cranial cavity, the largest velocities are found in the cerebral aqueduct, which is a narrow passage of length *L* ~ 10 − 15 mm and average radius *a* ~ 1 − 1.5 mm connecting the third and fourth ventricles [1,12]. These velocities have clinical potential value in investigating the development of NPH [6,13] and understanding the effects of different treatments like shunting [14] or lumbar puncture [15]. A closely related parameter of clinical interest is the transmantle pressure, or instantaneous pressure difference Δ*p*(*t*) between the lateral ventricles and the cerebral SAS. The largest pressure drop occurs across the aqueduct [16, 17] so that to a good approximation one can use the interventricular pressure difference to characterize the transmantle pressure difference, i.e. the pressure difference between the third and fourth ventricles Δ*p*(*t*) = *p*_3_ − *p*_4_. The value of Δ*p*(*t*) fluctuates from positive to negative about a negligibly small time-averaged value [18]. The pressure fluctuations, on the order of a few Pascals, drive the oscillatory flow across the aqueduct, with associated volumetric rates *Q*(*t*) having peak values on the order of 0.1 mL/s [19–21].

The transmantle pressure has been argued to play a role in the ventricle enlargement characterizing NPH patients [22]. Although the mean transmantle force exerted on the brain is very small [18], the cyclic strain field acting on the brain parenchyma over a long time can explain observed changes in brain viscoelastic properties in NPH patients with altered CSF flow dynamics [23]. The phase lag between Δ*p*(*t*) and the flow rate *Q*(*t*) has been investigated because of its potential clinical relevance in NPH patients [24], thereby underscoring the implications of the wave forms involved in aqueduct flow.

Direct measurements of the transmantle pressure require accurate simultaneous readings from two separate high-resolution pressure sensors [25], an invasive procedure with considerable risk factors [26]. Since the interventricular pressure difference Δ*p*(*t*) drives the flow in the aqueduct, the transmantle pressure can be inferred from noninvasive MRI measurements of the oscillatory aqueduct flow rate *Q*(*t*) [17], with the pressure differences associated with the cardiac-driven flow being about four times larger than those associated with respiration [25]. Different models have been developed for the relation between Δ*p*(*t*) and *Q*(*t*) [17,27,28], needed to enable estimates of the former from measurements of the latter [29]. As shown previously [28], accurate predictions require consideration of effects of flow acceleration as well pressure losses at the nearly inviscid entrance regions connecting the aqueduct with the ventricles.

In-vivo investigations of aqueduct flow using MR techniques have been instrumental in quantifying associated flow rates and stroke volumes (see, e.g. [9, 11] and references therein). More detailed quantitative information has been acquired in different computational investigations [30–33]. By way of contrast, in-vitro experimental studies of aqueduct flow are scarce. Particle image velocimetry has been used to characterize the velocity field induced by a syringe pump in an anatomically correct model of the cerebral aqueduct and the third ventricle [34]. Although interesting flow features were identified, the results correspond to steady flow, which is not representative of the markedly unsteady conditions found in the human aqueduct, for which resulting Strouhal numbers are of order unity [28]. Benchtop aqueduct experiments of unsteady flow were conducted by Holmlund et al. [35] using a simplified aqueduct model along with a realistic flow rate driven by a syringe pump. Corresponding interventricular pressure differences were measured and associated time-averaged values ≲ 0.5 Pa were computed, but corresponding pressure fluctuations and relative flow-rate/pressure-gradient phase lags, both of clinical interest, were not reported.

The purpose of the present in-vitro experiments is that of complementing the previous study [35] by providing a more complete description of the interventricular pressure difference for a range of flow conditions that match those found in healthy subjects. Experiments were conducted with an anatomically correct model, conveniently scaled up by a factor of 10, as needed to enable pressure measurements to be made, with care taken to maintain physical similarity. While most experiments considered a realistic time-dependent flow rate *Q*(*t*), to investigate influences of canal shape and flow-rate anharmonicity an initial set of measurements used instead a sinusoidal flow rate in an aqueduct model of constant circular section. The experimental results were validated through comparisons with predictions obtained using the previously published theoretical model [28]. The experimental measurements, presented in dimensionless form, provide a thorough description of the relation between the flow rate and the interventricular pressure difference, thereby enabling future transmantle pressure quantification based on non-invasive phase contrast (PC) MRI measurements. Our experimental approach allows for future the investigation of altered aqueduct CSF dynamics, such as that encountered in NPH patients.

## Methods

### Geometrical and physical similarity

To investigate the connection between the flow rate and the interventricular pressure, one could design an experiment mimicking exactly the cerebral aqueduct conditions. Water would be used as working fluid, since its density *ρ* = 1,000 kg/m^3^ and kinematic viscosity *ν* = 0.71 × 10^−6^ m^2^/s differ from those of CSF by a negligibly small amount. The setup would involve a tiny canal whose shape and size would have to be identical to those of the human aqueduct (i.e. *L* ≃ 10 mm and *a* ≃ 1 mm), a micro-pump able to deliver oscillating flow rates with peak values on the order of 0.1 mL/s, and a sensor with the capability of measuring pressure differences of a few Pascals with sufficient accuracy. Of course, such an experiment would be impractical, both because fabricating and handling such a small model would be challenging and because the devices that are required to deliver/measure accurately such small flow rates/pressure differences are uneconomical. The alternative procedure followed here involves scaling up the aqueduct model as well as the resulting flow rate and pressure difference, in such a way that the experiment can be performed in the lab using the available resources. The purpose of this section is that of describing the guidelines followed in the design.

To guarantee that the results of the experiment represent the flow in the human aqueduct, the experimental model must be geometrically similar to the human aqueduct, that is, its shape must be identical to that found in the human brain, but its size could be larger, thereby facilitating the experimental manipulation. Also, the experimental conditions must be physically similar to those found in the human aqueduct, in that all dimensionless governing parameters must take the same values in the experiment and the human brain. In reducing the parametric dependence of the interventricular pressure Δ*p*(*t*) we begin by noting that the oscillatory flow depends on the canal shape, including its length *L* and mean radius *a*, the density *ρ* and kinematic viscosity *ν* of the fluid, the frequency of the oscillatory motion (given, for example, by the angular frequency *ω*, related to the period *T* according to *ω* = 2*π/T*), and on the stroke volume $V_{s}=\frac{1}{2}\int_{t}^{t+2\pi /\omega }|Q|\text{d}t$ being displaced back and forth along the canal, which can be alternatively represented by the stroke length *L*_*s*_ = *V*_*s*_*/*(*πa*^2^). Following our previous analysis [28], we choose to characterize the pressure difference Δ*p*(*t*) by the mean value of its magnitude

(1) $$\langle |\Delta p|\rangle =\frac{\omega }{2\pi }\int_{t}^{t+2\pi /\omega }|\Delta p|\text{d}t,$$

so that we can write

(2) $$\langle |\Delta p|\rangle =\langle |\Delta p|\rangle \left(L,a,\rho ,\nu ,L_{s},\omega \right).$$

Since the functional dependence features six parameters, three of which have independent dimensions, straightforward application of the Buckingham Π theorem affords reduction from six dimensional parameters to three dimensionless parameters, leading to

(3) $$\langle |\Pi |\rangle =\frac{\langle |\Delta p|\rangle }{\rho \omega^{2}LL_{s}}=f\left(\frac{a}{L},\frac{L_{s}}{L},\alpha \right),$$

where

(4) $$\alpha ={\left(\frac{\omega a^{2}}{\nu }\right)}^{1/2}$$

is the Womersley number of the flow. The characteristic value used to scale Δ*p* follows from assuming that the local acceleration *∂u/∂t* ~ *L*_*s*_*ω*^2^ is comparable to the pressure force per unit mass *ρ*^−1^*∂p/∂x*, that being always the case for the flow conditions found in the cerebral aqueduct. While the aqueduct aspect ratio is very large, giving values of *a/L* in the range 1*/*20 ≲ *a/L* ≲ 1*/*10, the dimensionless stroke length *L*_*s*_*/L* and the Womersley number *α* are of order unity, with typical values lying in the ranges

(5) $$0.5≲L_{s}/L≲1.5\text{ and }2≲\alpha ≲4.$$

**Figure 1:**
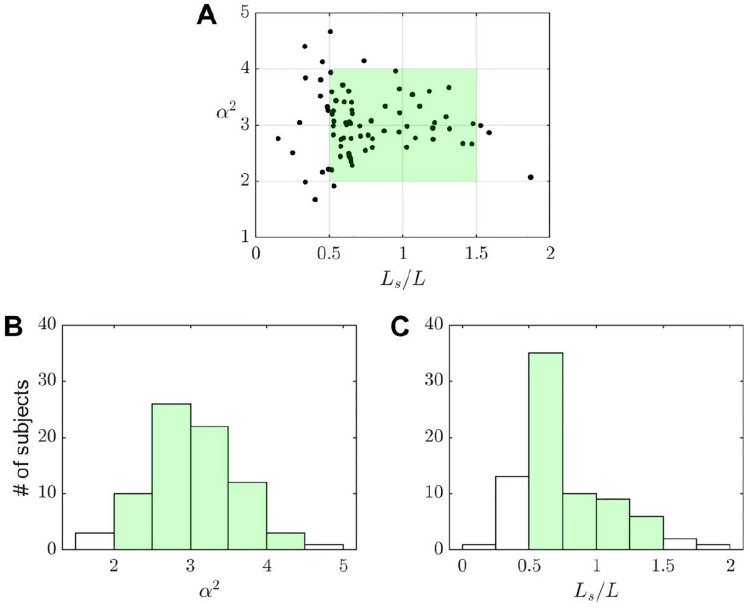
A) Scatter plot of values of the Womersley number (*α*) and stroke length-to-aqueduct length ratio (*Ls/L*) for 77 healthy volunteers participating in an IRB-approved study at HMRI, as reported elsewhere [29]. Distribution of these values for B) *α* and C) *Ls/L*.

For illustrative purposes, the specific values corresponding to the 77 subjects considered in our previous study [29] are shown shown in Fig. 1. The experiments reported below correspond to *α* = (2, 3, 4) with 0.5 ≲ *L*_*s*_/*L* ≲ 1.5, thereby covering the conditions most commonly found in healthy human subjects.

Regarding the design of the experiment, it is convenient to consider two flow configurations involving canals of different size that are geometrically similar, so that their aspect ratios *a/L* are identical. According to (3), if the values of *α* and *L*_*s*_*/L* are equal in both configurations, then the resulting values of the dimensionless pressure-fluctuation amplitude 〈|Π|〉〈= |Δ*p*|〉*/*(*ρω*^2^*LL*_*s*_) would also be equal. Therefore, to guarantee physical similarity, the dimensions, frequency, stroke length, and kinematic viscosity of the experiment (denoted by the subscript *E*) must be related to those of the human aqueduct (denoted by the subscript *H*) by

(6) $$\frac{a_{E}}{L_{E}}=\frac{a_{H}}{L_{H}},\;\frac{\omega_{E}a_{E}^{2}}{\nu_{E}}=\frac{\omega_{H}a_{H}^{2}}{\nu_{H}}\text{ and }\frac{L_{s,E}}{L_{E}}=\frac{L_{s,H}}{L_{H}}.$$

When the above relations are satisfied, then the interventricular pressure difference 〈|Δ*p*|〉_*H*_ can be computed in terms of the pressure difference 〈|Δ*p*|〉_*E*_ measured between the containers (representing the third and fourth ventricles) with use made of

(7) $$\frac{{\langle |\Delta p|\rangle }_{E}}{\rho_{E}\omega_{E}^{2}L_{E}L_{s,E}}=\frac{{\langle |\Delta p|\rangle }_{H}}{\rho_{H}\omega_{H}^{2}L_{H}L_{s,H}}.$$

The above expressions were used in scaling up the experiment by a factor *χ* = *L*_*E*_*/L*_*H*_ = *a*_*E*_*/a*_*H*_ = 10. In particular, the working fluid was selected to give experimental pressure differences 100 Pa ≲ Δ*p* ≲ 1500 Pa well within the operating range ±10 inH20 (±2490 Pa) of the available pressure sensor. The need for a working fluid different from water is apparent when using (6) to write (7) in the form

(8) $$\frac{{\langle |\Delta p|\rangle }_{E}}{{\langle |\Delta p|\rangle }_{H}}=\frac{\rho_{E}}{\rho_{H}}{\left(\frac{\omega_{E}}{\omega_{H}}\right)}^{2}\frac{L_{E}}{L_{H}}\frac{L_{s,E}}{L_{s,H}}=\frac{\rho_{E}}{\rho_{H}}{\left(\frac{\nu_{E}}{\nu_{H}}\right)}^{2}\chi^{-2}.$$

Clearly, in order for the pressure differential of the experiment with *χ* = 10 to be larger than that of the cerebral aqueduct (i.e. 〈|Δ*p*|〉_*H*_ ~ 5 Pa), the working fluid in the experiment must be significantly more viscous than CSF, thereby motivating the use of mixtures of glycerol and water. The experiments reported below, conducted in a temperature-controlled room at 21.5° C, employed three different glycerol-water mixtures with relative volume contents (84*/*16, 80*/*20, 74*/*26), yielding *ν*_*E*_ = (10.5, 6.82, 3.83) × 10^−5^ m^2^/s and *ρ*_*E*_ = (1225.6, 1216.4, 1202.2) kg/m^3^.

### Experimental setup

**Figure 2:**
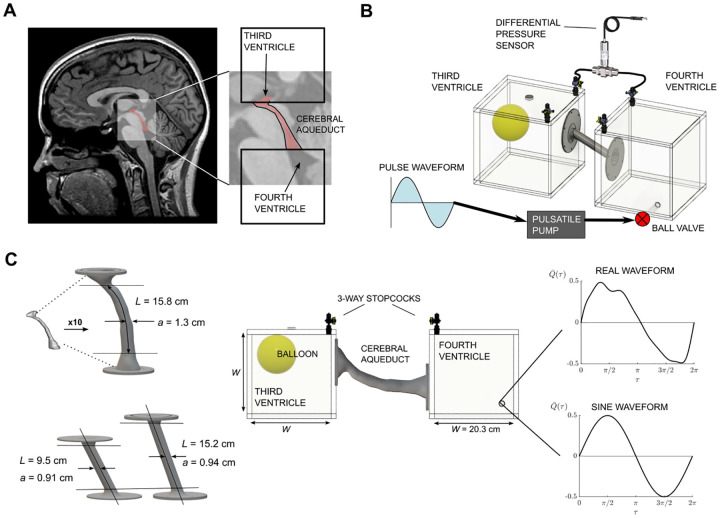
A) Sagittal MR image of the brain with segmented partial 3rd and 4th ventricles with the aqueduct (red) in the inset. B) Orthographic view of the experimental setup used to mimic the aqueduct flow. C) Geometries of the cerebral aqueduct with the corresponding geometrical parameters (real geometry - top left - and canonical geometries - bottom left), side view of the full experimental facility (middle) and experimental flow rate waveforms used as desired output from the pump (sine waveform - bottom right - and MRI waveform - top right).

As shown in the schematics of Fig. 2B and 2C, the in-vitro model consists of two large reservoirs of cubic shape connected by the cerebral aqueduct model. The reservoirs are constructed using acrylic sheets and assembled with acrylic cement. The periodic flow was generated with a programmable piston pump (SuperPump AR, ViVitro Labs, Victoria, Canada) connected through semi-rigid tubing to a ball valve located near the bottom of one of the reservoirs. The reservoir inner volume ~ 8 L is sufficiently large to minimize the pressure disturbances introduced by the associated intermittent jet stream. A latex balloon partially filled with air was included in the second reservoir to allow for compliance.

A first set of experiments employed the canonical aqueduct model shown in Fig. 2B, involving a circular cylinder of uniform radius whose edges were rounded to avoid flow separation during inflow. This simple configuration allowed us to test the dependence of the results on the aspect ratio *a/L* by using two tubes with different aspect ratios *a/L* = 0.097 and *a/L* = 0.062. A second set of experiments employed a realistic model of the cerebral aqueduct, shown in Fig. 2C, corresponding to the subject-specific anatomy used in our previous study [29] (associated IRB approved through Huntington Medical Research Institutes). In characterizing the subject’s anatomy, high-resolution anatomic MRI images were used to segment the CSF contained in the aqueduct using ITK-SNAP (Version 3.6.0; www.itksnap.org) [36]; i.e. see Fig. 2A. The resulting segmentation model was then scaled by a factor of 10 (*χ* = 10) and smoothed (Autodesk Meshmixer); i.e. see Fig. 2C. The segmentation represents the lumen of the cerebral aqueduct and was offset by 3 mm to create a hollow model with sufficient wall thickness to provide structural rigidity. After scaling up the model, the resulting inner mean radius and length were *a* = 1.3 cm and *L* = 15.8 cm, respectively, yielding an aspect ratio *a/L* = 0.082. The length of the real geometry was determined from our previous study, where the length is based on an increased of lumen size of 50% [29]. The scaled model printed for this study included some additional portion of the third and fourth ventricles seen in the red highlighted segmentation of the MRI image in Fig. 2. A detailed view of the canal geometry with indication of the resulting canal length is included as an inset in Fig. 5A.

For both geometries the connecting elements, representing the aqueduct, were 3D-printed on a Form 3 Stereolithography (SLA) resin desktop printer using clear resin (Formlabs). Printed parts were post-processed in accordance with the manufacturer instructions. The longer geometries were printed in two separate pieces due to the size limitation of the printer. Following post-processing, both pieces were joined using UV resin welding and epoxy resin after full cure. The models have flange attachments with a silicone o-ring which are secured to the reservoirs by screws and liquid PTFE (teflon) to avoid leakage.

As mentioned above, the experiments were performed using as working fluid mixtures of glycerol and water at different concentrations, resulting in different kinematic viscosities, as needed to establish the periodic motion with the desired values of *α*. A single viscosity solution would not have been able to accommodate the limited range of the pump frequency, stroke volume and pressure sensor limits (the mathematical basis for the selection of the mixtures is presented in a separate document [see Additional File 1]). Because of the large viscosity of the working fluid, additional care was needed when filling and purging the system to avoid trapped air bubbles in the glycerol-water mixture.

The periodic volumetric flow rate *Q*(*t*) considered in the experiments included a simple sine wave $Q=\frac{1}{2}V_{s}\omega \text{ sin}(\omega t)$ and a physiologically correct waveform determined using PC-MRI measurements in a healthy 25-year-old female subject [29]. In generating the periodic signal, the pulsatile pump uses as input the prescribed piston position, a zero-averaged signal that must start and end at the same value. The harmonic waveform is supplied as one of the default options in the pump software. For the realistic waveform, the piston position was determined using a trapezoidal cumulative numerical integration of the PC-MRI flow rate signal, and the result was scaled according to the manufacturer instructions to ensure the correct performance of the pump. The flow rate is adjustable within the limits of the pump, including frequencies in the range 3–200 BPM and stroke volumes in the range 0–180 mL/stroke.

### Data acquisition

A wet/wet differential bidirectional pressure transducer (OMEGA Engineering) connected to the top of the reservoirs was used to take measurements of Δ*p*(*t*) (device specifications can be found in Table 1). Although the connecting tubes were checked to be bubble free, complete elimination of air bubbles within the pressure transducer and/or pump was not possible in some instances, leading to pressure-measurement errors resulting from air compressibility. The pressure difference Δ*p*(*t*) was measured with a sampling frequency of 80 Hz. To synchronize Δ*p*(*t*) with *Q*(*t*) simultaneous recordings of instantaneous piston position were acquired using the pump’s voltage output. An Arduino Mega micro-controller, with an internal 10 bit ADC, was used to convert the voltage output to a digital signal, which was transmitted via a serial connection to a portable computer, where an open source serial data logger was used to time-stamp and save the data. The voltage was converted to piston position according to the manufacturer’s manual instructions. The sampling frequency of the analog output is 2500 Hz. The differences observed between the prescribed input piston position and the piston position computed from the pump’s voltage output were smaller than 5%, in agreement with the manufacturer’s stated waveform accuracy (i.e. < 4% of stroke volume at 70 BPM and < 5% of stroke volume at 200 BPM).

An experiment using a given glycerol-water mixture started by setting the corresponding amplitude-normalized wave form. Frequency values were selected in the range 0.15 Hz≤ *ω/*(2*π*) ≤ 1.17 Hz to provide the desired value of $\alpha ={\left(\omega_{E}a_{E}^{2}/\nu_{E}\right)}^{1/2}$, with results shown below for *α* = (2, 3, 4). With the pump running at constant frequency, pressure measurements were taken in subsequent tests for increasing values of the stroke volume *V*_*s*_ = *πa*^2^*L*_*s*_, an input variable that could be adjusted manually to cover the range 0.5 ≤ *L*_*s*_*/L* ≤ 1.5 in increments of Δ(*L*_*s*_*/L*) = 0.1. Each test comprised a total of 40–50 cycles. A complete list of measurements obtained is given in Table 2.

All signal processing was performed using a custom routine in MATLAB (Version R2019a; MathWorks). Signals were aligned using the timestamps of each recording. The pressure signal was filtered to eliminate noise and then zero-averaged. Pressure measurements from the sine wave flow rate were low-pass filtered at five times the selected frequency *ω*_*E*_, while the results corresponding to the subject-specific waveform were low-pass filtered at eight times *ω*_*E*_, as needed to retain sufficient high-frequency content. The piston-position signal, needed to synchronize the flow rate with the pressure difference, is subsampled to the same frequency as the pressure signal (80 Hz). This signal reading would produce some cases with sudden spikes much larger than the average peak value (>100x) which were detected using a Hampel filter and smoothed. The piston-position signal was differentiated to obtain the flow rate that was then low-pass filtered. The results, appropriately nondimensionalized according to

(9) $$\Pi (\tau )=\frac{\Delta p}{\rho_{E}\omega_{E}^{2}L_{E}L_{s,E}}\text{ and }\bar{Q}(\tau )=\frac{Q}{\omega_{E}\pi a_{E}^{2}L_{s,E}},$$

where *τ* = *ω*_*E*_*t*, were compared with predictions obtained using a previously published flow model [28]. Time-averaged magnitudes of the pressure difference $\langle |\Pi |\rangle =\int_{0}^{2\pi }|\Pi |\text{d}\tau /(2\pi )$ were calculated using the trapezoidal rule.

The definition of the phase lag between Π(*τ*) and $\bar{Q}(\tau )$ is not unique. One could for example define the phase lag based on the relative position of the maxima or minima of the two signals within a given cycle, but with this definition the resulting values were found to be very sensitive to changes in the waveforms. A more robust approach, employed here, involves use of the spectral domain. Thus, Fast Fourier Transform (FFT) was used to determine the fundamental mode and its corresponding phase for each variable, i.e.

$$ℱ[(\bar{Q}(\tau ))]\left(\omega_{0}\right)=\left|\bar{Q}\left(\omega_{0}\right)\right|e^{i\varphi Q}\;ℱ[(\Pi (\tau ))]\left(\omega_{0}\right)=\left|\Pi \left(\omega_{0}\right)\right|e^{i\varphi_{\Pi }},$$

with the phase lag determined from the difference between both phases *φ* = *φ*_Π_−*φ*_*Q*_. For definiteness, *φ* is defined in the range −*π* ≤ *φ* ≤ *π*, so that a positive value indicates that the pressure difference peaks before the flow rate.

## Results

**Figure 3:**
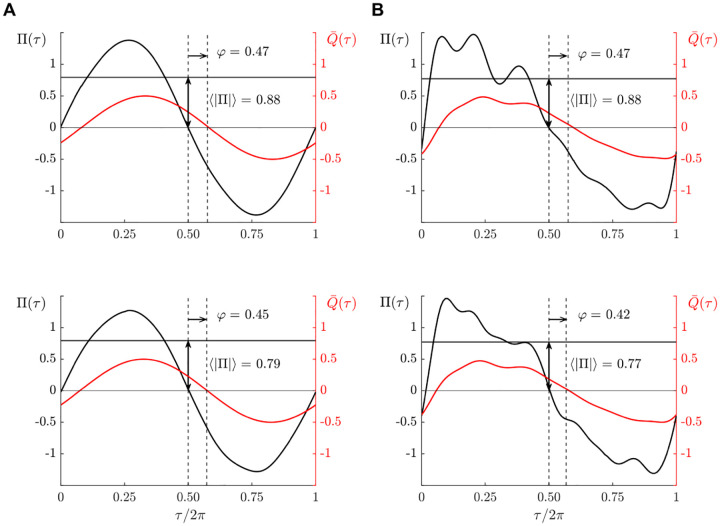
Dimensionless interventricular pressure Π(*τ*) corresponding to *α* = 2 and *L*_*s*_*/L* = 1 as determined from the previously published mathematical model [28] (top) and from the experimental measurements (bottom) for the cylindrical aqueduct (*a/L* = 0.062) with sinusoidal flow rate $\bar{Q}=\frac{1}{2}\text{sin }\tau$ (A) and with the PC-MRI flow rate (B). Each plot includes an indication of the associated time-averaged pressure difference 〈|Π|〉 and phase lag *φ*.

Pressure readings were obtained for the geometries and flow-rate signals indicated in Table 2. Resulting pressure waveforms corresponding to sinusoidal and physiologically correct flow rates are shown in Fig. 3 (bottom) for the canonical geometry, with corresponding model predictions shown (top). As shown in the left panels, for the harmonic flow rate, given by $\bar{Q}=\frac{1}{2}\text{sin}(\tau )$ in dimensionless form, the associated differential pressure is found to be nearly harmonic. By way of contrast, the physiologically correct flow rate induces a markedly anharmonic transmantle pressure Π(*τ*) exhibiting three peaks that are reminiscent of the percussion, tidal-wave, and dicrotic-wave peaks characterizing the intracranial pressure waveform [37]. The agreement between the experimental measurements and the theoretical predictions is quite satisfactory in all cases, thereby providing additional confidence in the description. For the particular case depicted in Fig. 3B, corresponding to *α* = 2 and *L*_*s*_*/L* = 1, the resulting time-averaged fluctuation amplitudes 〈|Π|〉 differ by approximately 14% and the corresponding phase lags *φ* exhibit differences of 12% from the model.

**Figure 4:**
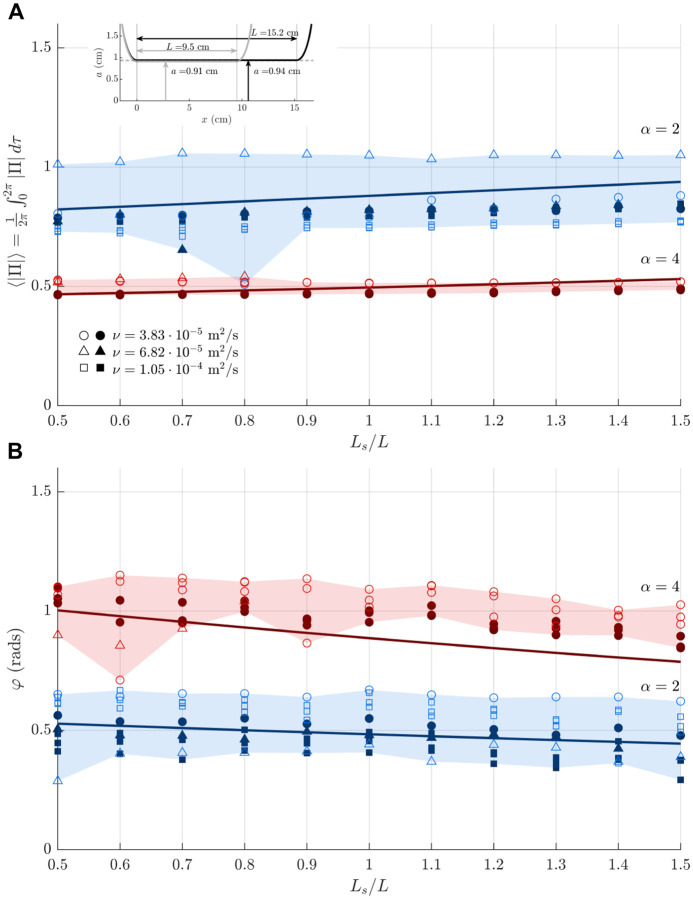
The variation with *L*_*s*_*/L* of the time-averaged magnitude of the pressure difference 〈|Π|〉 and phase lag *φ* for *α* = 2 (blue) and 4 (red) as measured with three different glycerol-water mixtures (viscosity values are indicated in the labels) in experiments using a harmonic flow rate along with a circular cylinder as a canonical representation of the aqueduct (hollow symbols: *a/L* = 0.096, solid symbols: *a/L* = 0.062).

Figure 4 represents dimensionless time-averaged pressure fluctuations 〈|Π|〉 and accompanying phase lags *φ* determined experimentally for the cylindrical configuration. Following the dimensional analysis presented earlier, the results are plotted for values of the reduced stroke length in the range 0.5 ≤ *L*_*s*_*/L* ≤ 1.5 and two different values of the Womersley number *α* = 2 (blue symbols) and *α* = 4 (red symbols). Results acquired for *α* = 3 are not included in the plot to avoid excessive cluttering [see Additional File 2 for additional results]. The plot includes measurements corresponding to three different glycerol-water mixtures, using two circular cylinders of different aspect ratio to represent the aqueduct, with results identified by either hollow markers (*a/L* = 0.096) or filled markers (*a/L* = 0.062). Theoretical predictions obtained with the mathematical model derived in [28] are represented by solid lines.

Except for an “outlier” set of experiments corresponding to the short geometry with *ν* = 6.82 × 10^−5^ m^2^*/*s (hollow triangles), possibly associated with the presence of non-visible small air bubbles in the system, the accordance of the different experimental results and the theoretical predictions is very satisfactory, especially with regards to the results for *α* = 4, thereby providing additional confidence on the selected parametric characterization of the flow. The analysis reveals that both 〈|Π|〉 and *φ* depend weakly on the dimensionless stroke length, variations remaining below about 10% over the entire range 0.5 ≤ *L*_*s*_*/L* ≤ 1.5 explored in the figure. By way of contrast, the dependence of the results on *α* is much more pronounced. As can be seen, as the Womersley number increases from *α* = 2 to *α* = 4, the pressure drop decreases by about 40%, that being a consequence of reduced effect of shear stresses, whereas the phase lag nearly doubles. The increase of *φ* with increasing *α* can be explained by noting that the phase lag between the interventricular pressure and the flow rate is mainly a result of the interplay of the different forces entering in the momentum equation. In flows at low Womersley numbers, there exists a balance between viscous and pressure forces, so that the associated flow rate *Q*(*t*) is in phase with Δ*p*. In contrast, in the opposite limit *α* ≫ 1, the flow rate and the interventricular pressure can be expected to be in quadrature, as follows from a balance between local acceleration and pressure gradient. The results in Fig. 4B are therefore consistent with the expected transition from *φ* = 0 (*α* ≪ 1) to *φ* = *π/*2 (*α* ≫ 1).

**Figure 5:**
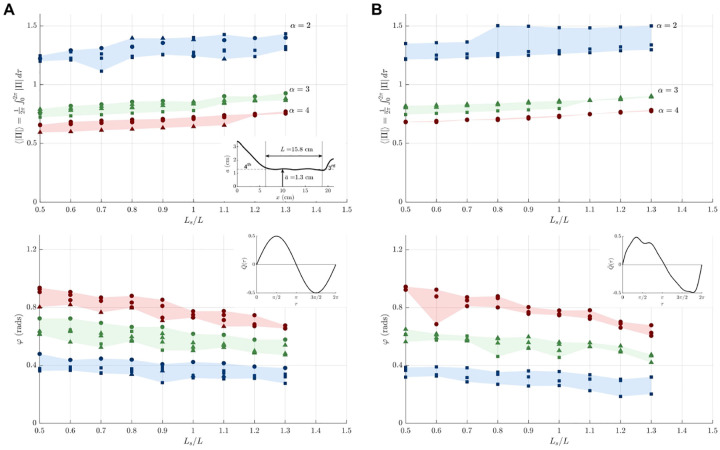
The variation with *L*_*s*_*/L* of the time-averaged magnitude of the pressure difference 〈|Π|〉 and phase lag *φ* for *α* = (2, 3, 4) as measured with three different glycerol-water mixtures (see labels in Fig. 4) in experiments using an anatomically correct aqueduct shape (top left inset) together with a harmonic flow rate (A) and an MRI-measured flow rate (B).

Measurements taken using the anatomically correct model are presented in Fig. 5 for both harmonic (Fig. 5A) and physiologically correct (Fig. 5B) waveforms *Q*(*t*). For this geometry, the large pressure variations associated with the experiments at large stroke volumes were found to compromise the structural integrity of the reservoirs, so that the experiments were restricted to values of *L*_*s*_*/L* ≤ 1.3. As in Fig. 4, very good agreement is obtained when the results of different working fluids are plotted in dimensionless form using *α* to characterize viscous effects.

The comparison between the top panels in Figs. 5A and 5B reveals that, while the waveform of the interventricular pressure is drastically different for the harmonic and physiologically correct flow rates, as seen in Fig. 3, the differences in the mean pressure fluctuation 〈|Π|〉 remain smaller than 5% for all values of *α* and *L*_*s*_*/L*. The differences in the phase lag are also very small, as seen in the lower plots, indicating that, for many quantitative purposes the flow rate can be represented by a simple sinusoidal function, as done in earlier analyses [17].

Effects of canal anatomy on the pressure drop can be assessed by comparing the results in the top panels of Figs. 4 and 5A, both corresponding to a sinusoidal flow rate. As can be seen, when written in dimensionless form, the interventricular pressure measured in the anatomically correct experiments is noticeably larger than that of the circular cylinders, a result attributable to the additional pressure loss occurring in the nozzle-like entrance regions of the third and fourth ventricles, which are included in the realistic model, as indicated in the inset of Fig.5A. In principle, the presence of these regions could be accounted for in defining the canal length, leading to larger values of *L* and correspondingly smaller values of 〈|Π|〉, as follows from its definition (3), thereby possibly improving agreement between the canonical and anatomically correct results.

## Discussion

Experiments employing a scaled physical model of the cerebral aqueduct have been used to characterize the relation between the flow rate *Q*(*t*) and the interventricular pressure difference Δ*p*(*t*). The focus has been on the time-averaged magnitude of the transmantle pressure 〈|Δ*p*|〉 and on the phase difference *φ* between Δ*p*(*t*) and *Q*(*t*), both quantities potentially having clinical interest in connection with NPH [23, 24]. Dimensional analysis was used to simplify the parametric dependence, leading to the reduced functional dependence identified in (3). A first set of experiments using two circular cylinders showed that the flow is fairly independent of the aspect ratio *a/L*, in agreement with previous theoretical results [23]. The dependences on the other two parameters (i.e. *L*_*s*_*/L* and *α*) are summarized in Figs. 4 and 5, with the results in the former figure validated through comparisons with theoretical predictions.

Of particular interest for quantitative purposes are the results shown in Fig. 5B, corresponding to an anatomically correct aqueduct geometry and a physiologically correct flow rate. The lower plot shows the phase difference *φ* between Δ*p*(*t*) and *Q*(*t*). As expected, the resulting value, weakly dependent on *L*_*s*_*/L*, is found to increase as flow acceleration becomes more pronounced with increasing *α*. On the other hand, the value of 〈|Π|〉 shown in the upper plot, can be used in (3) to provide

(10) $$\frac{\omega }{2\pi }\int_{t}^{t+2\pi /\omega }|\Delta p|\text{d}t=\langle |\Pi |\rangle \rho \omega^{2}L\frac{V_{s}}{\pi a^{2}},$$

which can be useful in computing the mean value of the transmantle pressure fluctuation from MRI measurements of the stroke volume *V*_*s*_ and the cerebral-aqueduct anatomy, the latter entering through the values of *L* and *a*.

The above expression is useful in discussing the clinical relevance of the stroke volume, a metric previously proposed as a predictor for shunt response in patients with normal pressure hydrocephalus [6]. Compared with normal subjects, NPH patients are known to have larger stroke volumes [38], which, according to (10), would be indicative of augmented interventricular pressure fluctuations, a reasoning that implicitly assumes that the aqueduct radius remains constant. However, enlargement of the aqueduct leads to a drastic reduction in the transmantle pressure, both because of the direct proportionality 〈|Δ*p*|〉 ∝ *a*^−2^ present in (10) and also because 〈|Π|〉 decreases with increasing $\alpha =a\sqrt{\omega /\nu }$, as shown in Fig. 5B. As a result, patients with simultaneous increased stroke volume and enlarged aqueduct crosssection may exhibit normal values of Δ*p*, as revealed in *in vivo* studies [19]. Clearly, future investigations addressing this issue can benefit from the simple quantitative description provided in (10).

Our experimental setup is the first attempt at an in-vitro characterization of the relation between aqueductal flow and interventricular pressure using an anatomically correct aqueduct geometry and a physiologically correct flow rate, thereby complementing previous experimental efforts [34,35]. The novel experimental setup allows for investigation of various input parameters (signal waveform, fluid viscosity, aqueduct geometry) allowing us to measure the corresponding effects on the differential pressure.

Future studies should consider the systematic variation of these parameters to understand certain diseases. Specifically, this experimental setup could be used to investigate the connection between changes in the frequency composition of the flow-rate signal, reported for example in [39], and the associated transmantle pressure. Also of interest are investigations of the relevance of the phase lag *φ* as a potential new marker of interest [24].

These future investigations could benefit from improvements to the experimental apparatus. For instance, because of the volumetric size and available compliance of the experimental setup, the experiments reported above were limited on the range of stroke volumes and frequencies applied to the system, with additional limitations arising from the available pressure transducer. A lower frequency and/or low stroke volumes incurred a larger noise contribution in the measurements. This was partially remedied through the use of different glycerol-water viscosity values and our attempt to balance the stroke volume and frequency. Additional sources of error include the presence of air bubbles within the main setup. We believe that a non-visible small air bubble likely within the pressure transducer and/or pump may be the cause for the outlier data points in the upper plot of Fig. 4.

The characterization of the phase lag on the basis of the fundamental frequency was found to be more robust than measurements based on cross-correlation or peak-to-peak time difference, which is partly due to the occurrence of signal jitter. However, this method relies on the availability of a large number of samples for high-frequency resolution, so that a decreased sampling frequency is needed for smaller time-sample sets. Future experiments should be recorded for longer duration to reduce this error. Additional systematic errors induced by the time-stamp alignment, which is on average about 1 ms (< 5% error), were minimized by sampling over many cycles and performing repeated experiments.

## Conclusion

Dimensional analysis has been used to guide the design of a scaled facility to investigate the flow in the cerebral aqueduct. MRI-informed in-vitro experiments have been used to evaluate the pressure difference between the third to fourth ventricles in the brain Δ*p*, yielding results in good agreement with those of a previously derived mathematical model. Mean pressure fluctuations have been characterized over parametric ranges describing the flow in the human aqueduct, leading to the derivation of a simple formula, given in (10), that facilitates quantification of the mean transmantle pressure from non-invasive MRI-measurements. The phase lag between the Δ*p*(*t*) and the flow rate *Q*(*t*), a metric with potential clinical application [24], has been found to depend mainly on the flow frequency and aqueduct radius (i.e. through $\alpha =a\sqrt{\omega /\nu }$), while the dependence on the stroke volume is much weaker. The new experimental facility can find future application in studies involving diseases that result in altered cerebrospinal fluid flow in the aqueduct such as NPH.

## Figures and Tables

**Table 1:** Differential pressure transducer configuration

Name	Description
Transducer Material	316L Stainless Steel
Process fitting	1/4–18 NPT Female
Pressure type	Differential Wet/Wet Bidirectional
Range unit	in-H2O (4°C)
in-H2O	10
Pressure transducer output	USB (high-speed)
Pressure transducer accuracy	±0.08% B.S.L.
Electrical termination	Cable (2m, 6ft)
Temperature range	18 to 85°C (−0 to 185°F)
Thermal accuracy: Zero shift / Span shift	±0.80% / ±0.60%

**Table 2:** List of experiments

Canonical (short)			Canonical (long)		Real Geometry	
*Glycerol-water (84–16 volume) ν* = 1.05 · 10^−4^ *m*^2^/*s*						
*α*	*ω*/2*π* (BPM)	*L*_*s*_/*L*	*ω*/2*π* (BPM)	*L*_*s*_/*L*	*ω*/2*π* (BPM)	*L*_*s*_/*L*
**2**	**48**	**0.5–1.5**	**45**	**0.5–1.5**	**24**	**0.5–1.3**
3	-	-	-	-	53	0.8–1.1
4	-	-	-	-	-	-
*Glycerol-water (80–20 volume) ν =* 6.82 · 10^−5^ *m*^2^/*s*						
2	31	0.5–1.5	29	0.5–1.4	15	0.8–1.1
**3**	**71**	**0.5–1.5**	**66**	**0.5–1.5**	**35**	**0.5–1.3**
4	126	0.5–0.8	-	-	62	0.5–1.1
*Glycerol-water (74–26 volume) ν =* 3.83 · 10^−5^ *m*^2^/*s*						
2	18	0.5–1.5	17	0.5–1.5	9	0.5–1.3
3	40	0.5–1.5	37	0.5–1.5	19	0.5–1.3
**4**	**71**	**0.5–1.5**	**66**	**0.5–1.5**	**35**	**0.5–1.3**

List of experiments conducted for each geometry and viscosity value with corresponding frequency *ω/*(2*π*) (expressed here in beats per minute) and sampled range of dimensionless stroke length (increment Δ*L*_*s*_*/L* = 0.1). The *α* value determined for each mixture and where measurements were made 3 separate times is given in bold.

## Data Availability

All data is available from the corresponding author upon request.

## Abbreviations

CSF: Cerebrospinal Fluid
NPH: Normal Pressure Hydrocephalus
PC-MRI: Phase contrast Magnetic Resonance Imaging
